# Acute Onset of Mesoamerican Nephropathy in a Patient With Chronic Gout and Occupational Exposure: A Case Report

**DOI:** 10.7759/cureus.80820

**Published:** 2025-03-19

**Authors:** Isabella Korchnoy, Juan Gabriel Jimenez Garcia, Guillermo Izquierdo-Pretel

**Affiliations:** 1 Internal Medicine, Jackson Memorial Hospital, Miami, USA

**Keywords:** chronic kidney disease, mesoamerican nephropathy, nephrotoxic, risk factor-based diagnosis, tubulointerstitial nephritis

## Abstract

Mesoamerican nephropathy (MeN) is a form of chronic kidney disease that is becoming increasingly prevalent. Multiple risk factors, including male gender, exposure to pesticides, heat stress, use of nephrotoxic medications, and a geographical association with Central America, have been linked to this condition. We present the case of a 40-year-old male from El Salvador who sought medical attention due to abnormal laboratory results and a flare-up of gout. The patient exhibited elevated creatinine and blood urea nitrogen (BUN) levels, with a significantly reduced glomerular filtration rate (GFR). His renal function had been stable three months earlier, when he had established care with his primary care physician. The patient had a history of agricultural work in El Salvador, along with long-term use of colchicine and nonsteroidal anti-inflammatory drugs (NSAIDs) for gout flare-ups. Following consultation with nephrology, he was diagnosed with MeN due to his relevant risk factors and clinical presentation. This case highlights the acute onset of MeN in a patient with established risk factors.

## Introduction

Mesoamerican nephropathy (MeN), also known as mesoamerican endemic nephropathy or chronic kidney disease of unknown cause (CKDu), was first identified in 2002 following a report from El Salvador that highlighted cases of chronic kidney disease (CKD) in young male agricultural workers [1]. Mesoamerica, a region in Central America encompassing Southeast Mexico, Guatemala, El Salvador, Western Nicaragua, and Northwestern Costa Rica, has experienced a growing burden of CKD cases with no identifiable cause. The emergence of this disease has garnered significant attention as a major public health issue in the region. Data from El Salvador and Nicaragua indicate that MeN is now the leading cause of premature death among young males [2,3].

MeN differs from other forms of CKD in that it is not associated with traditional risk factors such as diabetes or hypertension, which are common in other renal diseases. In the absence of a definitive cause, it is believed that multiple factors, such as environmental exposures, occupational hazards, and socioeconomic conditions, contribute to the development of MeN [2,4]. The diagnosis of MeN is one of exclusion, made when key risk factors are present and other known causes of CKD have been ruled out.

MeN predominantly affects young men from impoverished rural areas along the warm Pacific coast of southern Mexico and Central America, which explains its geographical concentration [5]. These communities endure harsh working conditions, significant heat exposure, environmental toxins, and the frequent use of agrochemicals [5-10]. Additionally, the widespread use of over-the-counter pain relievers and repeated episodes of dehydration further exacerbate the risk [5,11-13]. Critical social determinants such as poverty, limited access to healthcare, malnutrition, low birth weight, and elevated levels of social violence also play a role in the disease's progression.

The pathogenesis of MeN is thought to involve tubulointerstitial nephritis, which progresses to glomerular hypertrophy and ischemia [8,14-16], eventually leading to interstitial fibrosis, as reported in several studies [6]. Currently, there is no standard treatment specifically for MeN, aside from therapies for nonproteinuric CKD of other origins. However, early intervention and preventive strategies may help reduce the disease burden [3].

Despite increasing awareness of MeN as a significant health issue, there remains a scarcity of reported cases and in-depth studies on this condition. In this report, we present the case of a 40-year-old male diagnosed with MeN, alongside a comprehensive review of this emerging disease.

## Case presentation

A 40-year-old male immigrant from El Salvador with a long-standing history of gout presented to the emergency department after receiving a phone call from his primary care physician regarding abnormal renal function labs. He was advised to visit the nearest emergency department for further evaluation. Upon admission, the patient underwent a workup for acute monoarthritis and acute-on-chronic kidney disease.

The patient reported that, three months earlier, he had seen a primary care physician for the first time, where routine labs had revealed decreased renal function. Despite being advised to follow up with a nephrologist, he had not been able to schedule an appointment. He stated that his medical history consisted primarily of recurrent gout attacks, which affected his left big toe (hallux), left ankle, and both elbows. He had been managing his gout flares with nonsteroidal anti-inflammatory drugs (NSAIDs) for several years but had been switched to colchicine daily after his abnormal renal function was identified.

His social history was notable for heavy alcohol consumption on weekends in the past, during which he consumed large amounts of alcohol, though he had been abstinent for the last three years. He also shared that he had worked in agriculture in El Salvador for many years before immigrating to the United States 11 years ago. He currently works in construction, primarily installing floor tiles.

On presentation, the patient’s primary complaint was severe pain in his left ankle, which had rendered him unable to walk or work for the past week. He described this as one of many similar gout episodes in that ankle. He denied a history of nephrolithiasis, oliguria, bilateral pedal edema, and flank pain.

Physical examination

The examination revealed a warm, edematous left ankle with a decreased range of motion and an inability to bear weight. The right ankle was normal. A point-of-care ultrasound (POCUS) of the left ankle revealed a moderate effusion, with hyperechoic debris suggestive of tophi and possible bony erosions.

**Figure 1:**
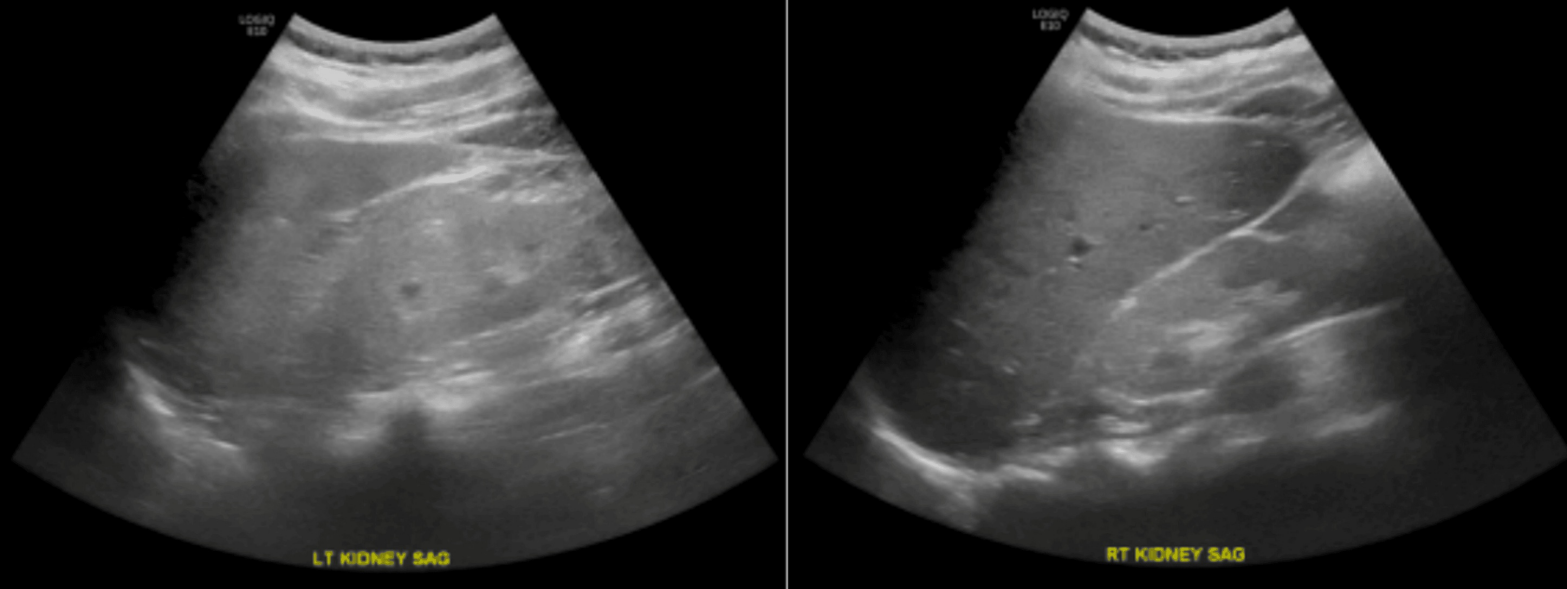
Renal ultrasound of the right and left kidneys showing kidney size with no appreciable hydronephrosis.

Renal ultrasound showed atrophic kidneys, likely due to long-standing kidney disease, with no appreciable hydronephrosis (Figure 1). His laboratory results are summarized in Table 1.

**Table 1:** Laboratory values ANA: antinuclear antibody; 25-OH: 25-hydroxy; HIV: human immunodeficiency virus; HCV: hepatitis C virus; N: normal

Test	Patient’s Values	Reference Range
ANA	1:80	N ≤ 1:80
Parathyroid hormone (PTH)	276 pg/mL	N = 10-65 pg/mL
Creatinine (Cr)	5.6 mg/dL	N = 0.6-1.2 mg/dL
Phosphorus	5.8 mg/dL	N = 3.0-4.5 mg/dL
Vitamin D 25-OH	6.0 ng/mL	N = 20-40 ng/mL
Blood urea nitrogen (BUN)	68 mg/dL	N = 7-20.0 mg/dL
Uric acid	9.0 mg/dL	N = 3.0-8.2 mg/dL
HIV and HCV tests	Negative	N = Negative
Urinalysis	Proteinuria	N ≤ 150 mg/day

Arthrocentesis of the left ankle was performed, yielding 2 cc of synovial fluid, which was sent for analysis. The synovial fluid was clear, with no crystals found, and a white blood cell (WBC) count of 1350 cells/µL. No bacterial growth was seen on culture. Given the elevated uric acid levels, a gout flare could not be excluded. The patient was treated with prednisone due to his stage V CKD, and colchicine was discontinued in light of his renal impairment.

The patient was discharged with a close follow-up planned with nephrology in the outpatient clinic. The patient progressed to end-stage kidney disease and started renal replacement therapy. He remains on chronic hemodialysis to date.

## Discussion

CKDu, also known as MeN, predominantly affects young agricultural workers in Central America [8]. While the precise etiology of MeN remains unclear, several risk factors have been identified. These include strenuous agricultural labor in hot climates with insufficient hydration; exposure to pesticides, agrochemicals, heavy metals, and metalloids; lower socioeconomic status; excessive use of NSAIDs; male gender; unidentified infections; low body mass index (BMI); consumption of sugary rehydration drinks; genetic predispositions; exposure to silica; and a family history of CKD [5,10-12].

Histopathological studies have attempted to better understand the presentation of this disease. Biopsies from previously healthy patients in Nicaragua who developed acute kidney injury (AKI) showed tubulointerstitial nephritis with varying degrees of inflammation and chronicity. Additionally, specimens from MeN patients in Nicaragua and El Salvador demonstrated the thickening of Bowman’s capsule and shrinkage of glomerular capillaries, suggestive of ischemic changes [3]. Another study investigating individuals at high risk of MeN in Nicaragua found biopsy results that consistently revealed tubulointerstitial nephritis with varying levels of chronicity and inflammation [3,14]. These findings suggest that tubulointerstitial nephritis may represent the early stages of MeN, while the ischemic changes reflect the disease's progression toward CKD.

There is currently no known treatment to halt or reverse the progression of MeN. However, a promising study published in December 2022 followed 18 patients over 36 months after they received a single injection of angiogenic/anti-fibrotic stromal vascular fraction (SVF) cells into the renal artery. At the two-month evaluation, increased perfusion to the interlobar/arcuate arteries was observed. After 12 months, patients with an initial estimated glomerular filtration rate (eGFR) >30 mL/minute were able to maintain their renal function, while those with lower eGFRs did not. The researchers hypothesized that the ischemic changes caused by glomerular fibrosis might be mitigated by improved vasodilation and perfusion, which could help salvage and repair affected glomeruli [6].

The high morbidity and mortality associated with MeN in endemic areas can be attributed to the lack of infrastructure to adequately treat these patients. Dialysis and kidney transplantation, which are essential treatments for advanced CKD, are often inaccessible to this population, exacerbating the healthcare crisis. It is crucial to improve access to these life-saving treatments in affected regions [3]. Preventive measures such as reducing heat exposure, ensuring proper hydration, and avoiding NSAIDs are practical steps to mitigate the progression of the disease.

Clinicians should consider MeN in patients from Central America who present with risk factors for the disease, a reduced eGFR, and no other identifiable cause of CKD [15]. Management of MeN primarily focuses on supportive care and preventing further disease progression. One potential preventive approach involves encouraging increased consumption of fluids containing sodium and potassium while avoiding sugary and fructose-laden beverages [16].

In our case specifically, we acknowledge the importance of considering alternative diagnoses in this patient with CKD, as MeN remains a diagnosis of exclusion. We recognize that the lack of a kidney biopsy limits diagnostic certainty and is a primary limitation. The patient's kidneys were small and echogenic on ultrasound, reflecting medical renal disease, and given the advanced stage and small kidney size, a kidney biopsy was not feasible. While this remains a key limitation of our case, the patient’s occupational and geographical history, along with his clinical presentation, strongly suggest MeN as the leading diagnosis.

It is important to discuss the several other diagnoses the nephrology team had considered to better understand how the diagnosis of MeN was established. Chronic urate nephropathy was a plausible differential, given the patient's long-standing history of gout and hyperuricemia. However, there was no evidence of urate crystal deposition or overt tubular dysfunction that would strongly suggest this diagnosis. Additionally, the patient's history of agricultural and construction work could potentially raise concern for environmental lead exposure, leading to chronic lead nephropathy. This was ruled out due to an absent history of early-onset gout and no laboratory evidence of lead toxicity (such as basophilic stippling of red blood cells or elevated lead levels).

Autosomal dominant tubulointerstitial kidney disease-UMOD (ADTKD-UMOD) was considered, given the patient’s early CKD onset, however, there was no reported family history of CKD or early-onset gout, which makes this diagnosis less likely. Of note, genetic testing was not performed. The chronic use of NSAIDs for gout flares could have contributed to chronic tubulointerstitial nephritis, causing analgesic nephropathy. However, the patient’s occupational history and geographical background, combined with his clinical course, favored MeN as the primary etiology. Other environmental and occupational exposures were explored, given the patient’s history of work in agricultural fields in El Salvador. Heavy metal exposure (cadmium, arsenic, pesticides) remains a consideration. However, no specific biomarker testing for these exposures was available.

The potential contribution of medication-induced nephrotoxicity has been addressed, as chronic NSAID use is a well-recognized cause of chronic interstitial nephritis. The nephrology team considered NSAID-induced kidney injury as a possibility but concluded that the patient’s prolonged occupational heat exposure and CKD pattern were more consistent with MeN. Alternatively, though nephrotoxicity from colchicine use is rare, we acknowledge its potential impact, but there were no systemic signs of colchicine toxicity in this case. Colchicine-induced toxicity was, therefore, ruled out as a potential cause.

Ultimately, the nephrology team considered NSAID-induced nephropathy versus MeN as the primary differential. The nephrology team evaluated potential reversible causes by stopping NSAIDs and optimizing hydration. In our patient, renal function did not improve despite hydration, suggesting an underlying chronic disease rather than an acute reversible insult. The patient’s laboratory data, urinalysis findings, and renal ultrasound all supported CKD. Additionally, there was no significant proteinuria to suggest glomerular disease. The patient did not have longstanding hypertension or diabetes to suggest hypertensive nephropathy or diabetic nephropathy, and all of these factors suggested chronic rather than AKI. After concluding that other etiologies of CKD were unlikely, MeN was determined to be the probable diagnosis.

## Conclusions

MeN is a diagnosis of exclusion that can be established based on a combination of clinical presentation and the presence of specific risk factors. Currently, there are no treatments proven to cure this condition effectively. SVF cell therapy has shown potential in improving renal perfusion and providing renoprotective effects in patients with an initial eGFR >30 mL/minute. To date, other than SVF therapy and the general management of CKD, no standard treatments have been demonstrated to effectively address this chronic and rapidly progressing disease. We present this case report to raise awareness and provide insight into the growing burden of this serious kidney disease, which currently lacks a standard treatment protocol.
